# First Complete Genome Sequence of Haematobacter massiliensis OT1 (Chromosome and Multiple Plasmids), Isolated from Human Skin

**DOI:** 10.1128/MRA.00292-19

**Published:** 2019-05-02

**Authors:** Jae Yun Lim, Munkhtsatsral Ganzorig, Shir-Ly Huang, Kyoung Lee

**Affiliations:** a Department of Agricultural Biotechnology, Seoul National University, Seoul, South Korea; b Department of Bio Health Science, Changwon National University, Changwon, South Korea; c Institute of Microbiology and Immunology, National Yang-Ming University, Taipei, Taiwan; Loyola University Chicago

## Abstract

Haematobacter massiliensis OT1 was isolated from human skin. This strain can catabolize 4-hydroxybenzoate. Here, we present the first complete whole-genome sequence of this species, which has one chromosome (2,467 kbp) and nine plasmids (total of 1,765 kbp). The analysis of the *H. massiliensis* OT1 genome indicated a potential for autotrophic growth.

## Abstract (precis)

Haematobacter massiliensis OT1 was isolated from human skin. This strain can catabolize 4-hydroxybenzoate.

## ANNOUNCEMENT

Haematobacter massiliensis is a Gram-negative aerobic bacillus belonging to the family *Rhodobacteraceae* (1). This species has been reported to be found on human skin and as a rare opportunistic pathogen linked with endocarditis and septicemia (1). Currently, only three genome assemblies of *H. massiliensis* are publicly available in the NCBI database, each having more than 50 scaffolds. Here, we report the complete genome sequence of *H. massiliensis* OT1, an isolate from human skin.

*H. massiliensis* OT1 (KCTC 62810) was isolated from the forehead of a male college student by swab sampling and direct streaking on minimal salts basal (MSB) medium containing 0.01% yeast extract and 0.05% 4-hydroxybenzoate (2). The agar medium was incubated for 3 days under aerobic conditions at 28°C for growth. Ethics approval for subject sampling was granted by the institutional review board of Changwon National University. Total DNA was extracted using the phenol extraction method from *H. massiliensis* OT1 cells cultured in the liquid medium under the conditions described above (3). The genomic SMRTbell library with a total of 5 μg was constructed with the SMRTbell template prep kit v1.0 (part number 100-259-100), following the manufacturer’s instructions (Pacific Biosciences). Fragments of the template smaller than 20 kbp were removed using the BluePippin size selection system, and the constructed library was validated using the Agilent 2100 Bioanalyzer. The SMRTbell library was sequenced using 1 single-molecule real-time (SMRT) cell using C4 chemistry, and 240-minute movies were captured for each SMRT cell using the MagBead OneCellPerWell v1 protocol (Pacific Biosciences) of the PacBio RSII sequencing platform (DNA Link, Seoul, South Korea). *De novo* assembly was conducted according to the Hierarchical Genome Assembly Process (HGAP) workflow v2.3, including consensus polishing with Quiver (4). After filtering the subreads, a pool of 1,656,433,062 bp from 152,503 long reads with an *N*_50_ value of 2,496,671 bp were produced, yielding 233-fold genome coverage. Complete genome sequences were obtained using bioinformatics analyses as previously described (5). Gene predictions and annotations were provided by NCBI using the NCBI Prokaryotic Genome Annotation Pipeline (6). The SEED subsystem was used via Rapid Annotations using Subsystems Technology (RAST) for functional categorization of the predicted proteins (7).

The complete genome of *H. massiliensis* OT1 contains one circular chromosome and nine plasmids, named pOT1-1 to pOT1-9. The genome statistics are shown in Table 1. It is interesting to note that the plasmids constitute 42% of the genes and contain one copy of rRNA genes (5S, 16S, 23S) and six coding regions of tRNAs. The genes encoding the enzymes that catabolize 4-hydroxybenzoate through the β-ketoadipate pathway are carried on the chromosome (*pcaIJF*) and plasmids pOT1-1 (*pcaD*) and pOT1-3 (*pobA* and *pcaCHGB*) (8). The *H. massiliensis* OT1 genome contains genes related to the Calvin-Benson cycle and the RuBisCO genes (*rbcLS*) for autotrophic growth, as found in Paracoccus yeei TT13, another skin isolate (9). The *H. massiliensis* OT1 genome will help reveal not only the cellular and catabolic adaptation mechanisms of this bacterium to colonize human skin but also the acquisition of multiple plasmids.

**TABLE 1:** General genomic features of *H. massiliensis* OT1

Genome	Genome size (bp)	No. of coding sequences	G+C content (%)	No. of rRNAs	No. of tRNAs	GenBank accession no.
Chromosome	2,467,987	2,375	64.72	6	42	CP035510
pOT1-1	398,426	348	64.53	0	1	CP035511
pOT1-2	349,143	301	64.63	3	4	CP035512
pOT1-3	285,811	250	64.45	0	1	CP035513
pOT1-4	187,343	158	65.33	0	0	CP035514
pOT1-5	183,422	172	61.61	0	0	CP035515
pOT1-6	118,868	95	64.90	0	0	CP035506
pOT1-7	103,683	94	64.42	0	0	CP035507
pOT1-8	83,590	88	62.38	0	0	CP035508
pOT1-9	54,639	53	61.84	0	0	CP035509
Total	4,232,912	3,934	64.48	9	48	

### Data availability.

The complete genome sequences, including those of plasmids of the *H. massiliensis* OT1 strain, were deposited in GenBank under the accession numbers CP035506 to CP035515 (Table 1). This genome has a base modification file available under GenBank accession number CP035510. The raw sequencing data have been deposited in the SRA under accession number PRJNA517385.
